# Two Different High Throughput Sequencing Approaches Identify Thousands of *De Novo* Genomic Markers for the Genetically Depleted Bornean Elephant

**DOI:** 10.1371/journal.pone.0049533

**Published:** 2012-11-21

**Authors:** Reeta Sharma, Benoit Goossens, Célia Kun-Rodrigues, Tatiana Teixeira, Nurzhafarina Othman, Jason Q. Boone, Nathaniel K. Jue, Craig Obergfell, Rachel J. O'Neill, Lounès Chikhi

**Affiliations:** 1 Population and Conservation Genetics, Instituto Gulbenkian de Ciência, Oeiras, Portugal; 2 Organisms and Environment Division, School of Biosciences, Cardiff University, Cardiff, United Kingdom; 3 Danau Girang Field Centre, Sabah Wildlife Department, Kota Kinabalu, Sabah, Malaysia; 4 Floragenex, Inc., Eugene, Oregon, United States of America; 5 Center for Applied Genetics and Technology, Department of Molecular and Cell Biology, University of Connecticut, Storrs, Connecticut, United States of America; 6 CNRS, Laboratoire Evolution and Diversité Biologique, Université Paul Sabatier, Toulouse, France; 7 Université de Toulouse, Toulouse, France; Natural History Museum of Denmark, University of Copenhagen, Denmark

## Abstract

High throughput sequencing technologies are being applied to an increasing number of model species with a high-quality reference genome. The application and analyses of whole-genome sequence data in non-model species with no prior genomic information are currently under way. Recent sequencing technologies provide new opportunities for gathering genomic data in natural populations, laying the empirical foundation for future research in the field of conservation and population genomics. Here we present the case study of the Bornean elephant, which is the most endangered subspecies of Asian elephant and exhibits very low genetic diversity. We used two different sequencing platforms, the Roche 454 FLX (shotgun) and Illumina, GAIIx (Restriction site associated DNA, RAD) to evaluate the feasibility of the two methodologies for the discovery of *de novo* markers (single nucleotide polymorphism, SNPs and microsatellites) using low coverage data. Approximately, 6,683 (shotgun) and 14,724 (RAD) SNPs were detected within our elephant sequence dataset. Genotyping of a representative sample of 194 SNPs resulted in a SNP validation rate of ∼ 83 to 94% and 17% of the loci were polymorphic with a low diversity (*H*_o_ = 0.057). Different numbers of microsatellites were identified through shotgun (27,226) and RAD (868) techniques. Out of all di-, tri-, and tetra-microsatellite loci, 1,706 loci had sufficient flanking regions (shotgun) while only 7 were found with RAD. All microsatellites were monomorphic in the Bornean but polymorphic in another elephant subspecies. Despite using different sample sizes, and the well known differences in the two platforms used regarding sequence length and throughput, the two approaches showed high validation rate. The approaches used here for marker development in a threatened species demonstrate the utility of high throughput sequencing technologies as a starting point for the development of genomic tools in a non-model species and in particular for a species with low genetic diversity.

## Background

The field of population genetics has been dominated for many years by the use of microsatellites which have been developed and tested across a wide range of species with enormous success [1], [2], [3], [4]. More recently, single nucleotide polymorphisms (SNPs) have been identified as potential markers of choice for genome-wide studies [5] due to the fact that they are broadly distributed in the genome (like microsatellites), have the advantage over microsatellites of being easily typed in large numbers, and represent variation in both coding and noncoding regions of the genome [6], [7]. The first applications have been limited to model organisms but there is a growing interest in rapidly developing large numbers of markers and applying them to non-model organisms [8], [9] to study evolutionary questions [10] and address conservation genetics issues [11]. SNPs should therefore allow geneticists to inspect both neutral variation and genomic regions under selection. They should also be useful to work with non-invasive or historical samples. Indeed, Morin & McCarthy [12] used 19 SNPs in a study using historical samples (bone and baleen) of bowhead whales; they found a 0.1% genotyping error rate, which is lower than most non-invasive studies often reported for microsatellite genotypes obtained from other samples (e.g. 0.8% from tissue, 2.0% from faeces [13]. The low error rate together with their binary nature suggests that it may be more feasible to genotype SNPs in highly degraded samples (hair, faeces, etc.) such as those obtained from endangered species [12], [14]. It also means that much of the traditional population genetics theory that was originally developed for bi-allelic markers will directly apply, whereas multiallelic markers have always generated debates on the best way to define classical measures of genetic diversity or differentiation, for instance [15], [16], [17].

A number of approaches have been used to discover *de novo* SNPs including the targeted gene approach using ‘CATS’ (comparative anchor tagged sequences) or ‘EPIC’ primers (exon priming intron crossing) [18], [19], [20], [21], [22], AFLP/RFLP [23], [24], and EST (Expressed Sequence Tag) sequencing [25]. However, most of these approaches, with the exception of ESTs, have only identified a limited subset of markers for genotyping (between 15 and 318 markers). Taking into account the fact that the number of SNP markers required for population genetic analyses is likely to be between 5 and 10 times that of microsatellites to reach the same statistical power (e.g., [26], [27]), none of these methods has yet proven that it had the potential to meet the requirements of future molecular studies [28].

The development of high throughput sequencing platforms has emerged as a new tool to develop genomic markers and is already showing great promise for fast and efficient marker detection for model and non-model species [28], [29]. These methods vary in their applicability in terms of research questions and molecular resources required. One strategy to develop new genomic markers might be to simultaneously sequence multiple genomes of the targeted organism and identify markers by comparing sequences from the total data set [30]. This method was used, for example, to identify 23,742 SNPs in the uncharacterized genome of *Eucalyptus grandis* [31]. However, sequencing and producing high quality reference genome (approx. 3 Gb genome size for a non-model mammal species) using shotgun genome sequencing is still an expensive and substantial project. Other alternatives to develop markers could be achieved by sampling and sequencing only a reduced but still large part of the genome, and may consequently be more affordable [32]. Examples of such approaches include the construction and sequencing of reduced-representation libraries (RRLs) and restriction site-associated DNA sequencing (hereafter RAD-seq, also known as RAD-tag) using the two most popular platforms, i.e. Roche 454 FLX (hereafter 454) and the Illumina Genome Analyzer (hereafter Illumina) [32]. RRLs have been employed for the discovery of thousands of SNPs in species for which a genome sequence is available, such as human [5], cattle [33], pig [34], and turkey [35], as well as in species for which a reference genome is not available such as the great tit [9], and mallard Duck [36]. The RAD-seq methodology consists of screening thousands of regions adjacent to restriction sites to subsample the genome [37]. This methodology has been applied in threespine stickleback to investigate (i) the genetics of an important trait (presence/absence of bony lateral plates) [37], (ii) population differentiation, and (iii) selection [38], and more recently to unravel the phylogeography of pitcher plant mosquito [39].

While high throughput SNP genotyping platforms can in principle rapidly and efficiently genotype many loci on hundreds of samples identifying SNPs that fit the criteria for assay development can be challenging in organisms without a reference genome. Two reasons are that SNP genotyping assays often require SNPs to have large windows free of nearby polymorphisms and SNPs should be evenly spaced throughout the genome [32], [40]. RAD-seq generates short DNA fragments (RAD-tags) that have one end defined by the restriction enzyme recognition site, and the other end defined by random shearing [41]. 454 sequencing technology on the other hand produces larger DNA sequences [28]. However, the power of these methodologies to discover markers in non-model species (i.e. with no reference genome) and especially in endangered mammals is still largely unexplored and remains a challenge. It is an open question whether RAD-seq and 454 shotgun sequencing will bring population genetics analysis of every organism into the high throughput sequencing age.

The Bornean elephant (*Elephas maximus borneensis*) is a subspecies of mainland Asian elephant which differs from other subspecies in its morphology and behaviour [42], [43], [44], [45]. Their distribution is restricted to the North of Borneo where the estimated population size is around 2,000 in Sabah [46]. Bornean elephants are classified as endangered according to the IUCN (International Union for Conservation of Nature) Red list of threatened species. The main threats identified are habitat fragmentation and habitat loss due to oil palm plantations. No microsatellite markers have been yet designed specifically for the Bornean elephant and the only known study by Fernando *et al*., [42] used five microsatellites originally developed for other Asian elephant subspecies. The authors genotyped fifteen individuals and found a very low level of genetic diversity, with three microsatellites being monomorphic, and the other two exhibiting only two alleles. No mitochondrial (mt) DNA polymorphism has yet been found in this species [42]. With such a low level of genetic diversity the Bornean elephant represents thus a very iconic species for which the development of polymorphic markers would be a potential example for many others. If markers can be developed here then it should be applicable to most other endangered species. Therefore, our aim was to (i) discover *de novo* markers (SNPs and microsatellites) for the Bornean elephant by using the advantages offered by two different high throughput sequencing methodologies (454 shotgun and Illumina GAIIx-local *de novo* assembly of RAD paired-end reads), (ii) develop a genotyping assay for a reasonably large subset of identified SNPs, and (iii) validate a subset of these markers on high quality (blood) samples.

By using the combination of shotgun and RAD-sequencing, we demonstrate that the chosen approaches represent effective strategies towards development of comprehensive molecular resources for this and other endangered species for which faecal samples are the only available samples to carry out conservation genetic studies.

## Results

### RAD Paired-end (Illumina) and 454 Shotgun Sequencing and Local *de novo* Assembly

More than ∼ 2.3 million sequence reads from each of the four elephant samples (sample 2, 3, 4 and 7) were used in local *de novo* assembly of RAD paired-end reads, which was equivalent to ∼ 300 Mb of *de novo* sequence generated per sample. All Illumina sequence data have been submitted to the EBI Sequence Read Archive (SRA) under the study accession number ERP001879 (http://www.ebi.ac.uk/ena/data/view/ERP001879). The four samples chosen for *de novo* assembly performed better than the other samples in terms of high output of sequence, coverage, quality (as inferred by phred quality score), and in terms of the total number of RAD-tags generated. The variability seen in read numbers observed for each sample was mostly due to the overall quality of the input material. Some samples were slightly more degraded than others. The number of sequenced RAD-tags obtained was highly dependent on the sequence amount and number of reads per cluster (i.e. the reads that can be aligned to each other and were grouped into pools representing the same RAD tag to build contigs) (Table 1). A breakdown of the number of sequence reads and all other detailed statistics obtained for each elephant sample is listed in Table 1. After removing any assemblies with too high (due to repetitive regions in the genome) and too low coverage, ∼2.0–2.5 Mb of high quality sequence was available for each of the four Bornean elephant samples. Based upon the observed amount of sequence for each sample, the estimated number of RAD-tags ranged between 23,532 (sample 6) and 37,362 (sample 7). Of the four samples that obtained the highest quality and quantity of sequence we assembled the following numbers of contigs: sample 2–10,008 contigs, sample 3–10,110 contigs, sample 4–10,352 contigs and sample 7–7,918 contigs. Since sample 4 had the highest quality sequence and the highest number of assembled contigs we used the contig set from sample 4 as a reference with which to align all other paired end sequences to identify SNPs.

**Table 1:** Summary statistics of data obtained using two different sequencing approaches: Illumina (RAD-seq) and 454 (shotgun).

Feature	Illumina RAD-sequencing								454 shotgun sequencing	
	samples (*n* = 8)								samples (*n* = 2)	
	1	2	3	4	5	6	7	8	A	B
Reads (millions)	1.84	2.32	2.54	2.59	2.17	1.05	2.53	1.26	0.51	0.64
Aligned reads between samples (%)	–	–	–	–	–	–	–	–	219,157 (19.17%)	
Mb of sequence	221.13	278	305.01	311.12	259.92	125.74	303.47	150.95	–	
Total number of RAD tags produced (approximate)	33,698	32,330	32,937	35,014	34,815	23,532	37,362	28,573	–	
Total Mb of sequence after contig construction	–	2.88	–	2.68	–	–	2.07	1.50	–	
Contigs assembled	–	10,008	10,110	10,352	–	–	7,918	5,461	16,857 (>100 bp)	
Average contig length (bp)	–	288	–	259	–	–	262	275	328.13	
Average depth of coverage per base of contigs	12.9x								8x	
N50*(bp)	–	320	–	279	–	–	281	302	815	
Contig length range(bp, min-max)	–	150–560	–	150–457	–	–	150–544	150–527	100–6,407	
Reads mapped to *L. africana*	–	–	–	–	–	–	–	–	497,169 (97.6%)	617,930 (97.2%)
Putative SNPs	14,724								6,683	
Total number of homozygotes (monomorphic)	9,676								–	
Total number of heterozygotes	5,048 (34%)								–	
Transitions and transversions(Ts/Tv ratio)	1.61								1.52	
Candidate loci containing SNPs	20%(2,100 out of 10,352)								10%(1,753 out of 16,857)	
Loci suitable for Sequenom assay with >q20# and identified assays	518 (24.6%),19								1,695 (96.6%), 52	
SNP density	0.00081								0.00056	
Validation of SNPs(genotyping success rate acrossfive plexes)	86–95%(plex1–plex4)								91%(plex5)	
Polymorphic loci(*n* = 194)	28 (*E.m.borneensis*), 17 (*E.m.indicus*)								5 (*E.m.borneensis*), 7 (*E.m.indicus*)	
Number of contigs containing microsatellite loci	837								18,195	
Number of SSRs identified(mono-,di-,tri, and tetra-nucleotides)	868(844 mono-, and 24 di-nucleotides)$								9,038(18,188,7,241, 1,471, 326)	
Potential amplifiable loci(with ≥3 repeats)	7 (29%)								1,706 (18.8%)	

Note that the eight elephant samples (1 to 8) used in Illumina RAD-sequencing are different from the two.

samples (A and B) used in 454 shotgun sequencing.

* weighted median statistic such that 50% of the entire assembly is contained in the number of contigs equal.

to or greater than this value.

# >q20: 0.01% chance that a base was wrongly called.

$ identified in elephant sample 4.

*De novo* contig lengths ranged from 150 (all samples) to 560 bp (sample 2) with mean length between 259 and 288 bp (sample 4 and 2). The average depth of coverage per base of contigs was 12.9x. The contigs assembled from each RAD-sequence had an N50 length between 279 and 320 bp (Table 1). N50 is the weighted median statistic such that 50% of the entire assembly is contained in the number of contigs equal to or greater than this value [47]. 2.68 Mbp of *de novo* sequence was assembled from sample 4 with an average contig length of 259 bp. In total, 14,724 SNPs were identified by mapping the raw reads from other elephant samples with this ‘mini-reference’ contig library from sample 4. Across all datasets, 5,048 (34%) SNPs were identified as heterozygotes (using the threshold parameter as implemented in SAMtools) and 9,676 as homozygotes (monomorphic). Altogether 20% (2,100 loci out of 10,352 contigs) were identified as variant loci. Out of these we obtained a genotype for all individuals (100%) for 1,200 loci/RAD tags, and for 75% of the individuals (i.e. six or more) for 1,700 loci (Table S1 provides the complete list). For the remaining 400 loci, the SNPs were shared by less than six individuals.

**Figure 1:**
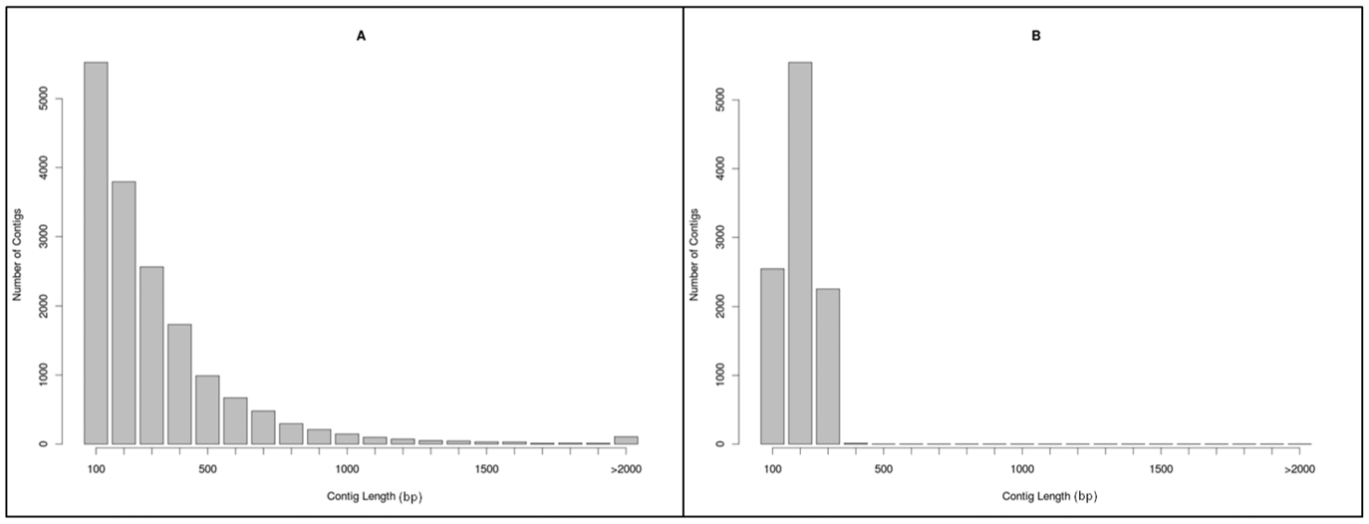
Histogram of contig size distribution. Length distribution of contigs assembled from 454 shotgun sequencing (A) and RAD-sequencing (B) using Illumina platform. The average contig length in the 454 dataset (*n* = 2) was 328.13 bp and ranged from 100 to 6,407 bp. The average contig length in the RAD-sequencing dataset (*n* = 8) was between 259 (sample 4) and 288 bp (sample 2). The RAD contig lengths ranged from 150 to 560 bp.

A full run on the Roche 454 GS-FLX system yielded a total of 1,144,367 reads for the two elephants used with elephant sample A and B generating 509,030 and 635,337 reads, respectively (Table 1). The average read length was 348 bp. The raw sequence data from 454 sequencing instrument have been submitted to the EBI Sequence Read Archive (SRA) under the study accession number ERP001879 (http://www.ebi.ac.uk/ena/data/view/ERP001879). Altogether 219,157 (19.17%) out of 1,144,367 reads aligned to each other from elephant sample A and B and were assembled into contigs. This was the total amount of data used for contig assembly. The total number of *de novo* assembled contigs (>100 bp) obtained was 16,857 with an average contig length of 328.13 bases. There were 3,267 large contigs (>500 bp) with an average contig size of 841 bases, with the largest contig size identified as 6,407 bp. The length distribution of the 454 and RAD-seq (Illumina) assembled contigs are shown in Figure 1. The assembled contigs in the 454 data set had an N50 length of 815 bp and average coverage per bp of all contigs was 8x. Sample A and sample B mapped 42.1% and 42.5% of their reads to this reference, respectively, resulting in about 42.4% of the reads utilized in SNP calling.

To evaluate the quality of the sequences, reads from 454 sequencing were mapped to the African elephant (*L. africana*) genome and 497,169 (97.6%) reads from sample A and 617,930 (97.2%) reads from sample B were successfully mapped (Table 1).

### Development of Genotyping Assay for Markers (SNPs and SSRs) Identified Using RAD-seq and 454 Shotgun Sequencing

In the RAD-seq data set, the total proportion of observed transitions in Bornean elephant (A–G = 380, C–T = 404; total = 784) was higher than that of transversions (A–C = 132, G–T = 115, A–T = 61, C–G = 178; total = 486), which corresponds to a ts/tv ratio of 1.6∶1. Our results showed that the number of A–G substitutions almost equaled the number of C–T substitutions in the transitions class. Moreover, the substitutions within the transversions class occurred in comparable frequencies, with the exception of A–T.

Filtering of the contigs containing SNPs in RAD-seq data set resulted in identification of 518 (24.6%) putative SNPs with phred quality score, >q20 (corresponding to a 0.01% chance that a base was wrongly called) out of the 2,100 variant loci with favorable characteristics for downstream genotyping design. 491 out of 518 loci were identified with quality score, >q30 (corresponding to a 0.001% chance that a base was wrongly called). The observed SNP density (number of SNPs/bp) was 0.00081(∼8/10 kb). These SNPs were identified in all eight Bornean elephant lines and were used for Sequenom assay design. We then eliminated 11 contigs because 10 of them were identified with a third allele (tri-allelic state) and one contig indicated primer design error. This left a total of 507 independent SNPs for further validation and characterization in 19 multiplexes (Table 1). In addition, we found candidate SNP mutations specific to each elephant that distinguished it from all others (minor alleles only found in one heterozygous individual). More specifically the numbers of these were 146, 164, 152, 139, 162, 133, 146, and 120 in sample 1 to 8, respectively. Given the sample size, these probably correspond to rare alleles which may be found in other non-sampled individuals, but may be useful to identify specific individuals using multi-locus SNP data.

Screening of 10,352 contigs from elephant sample 4 resulted in the identification of 868 SSR with 844 mono-, and 24 di-nucleotide repeats in 837 contigs (8% of total contigs) with 31 (3.7%) contigs that contained more than one type of repeat motif. 17 SSR were present in compound formation. Out of 24 di-nucleotide repeats, 7 loci (29%) with ≥3 repeats were identified as ‘potentially amplifiable loci’ (PAL). No tri-and tetra-nucleotide repeats were identified in this limited sequence data set from sample 4 (Table 1).

The shotgun genome sequencing of the two elephant samples resulted in identification of 18,206 variants (6,683 SNPs and 11,523 indels) residing in 5,885 contigs (i.e. 35% of the loci out of the total 16,857 assembled contigs). 1,753 (10%) contigs out of 16,857 contigs were identified to contain these 6,683 SNPs (Table 1). The calculated SNP density in 454 contigs was 0.00056 (∼5/10 kb) which is not much different from the SNP density found in RAD-seq. The total proportion of identified transition substitutions (A–G = 2020, C–T = 2010; total = 4030) were higher than transversions (A–C = 680, G–T = 628, A–T = 463, C–G = 886; total = 2657), which corresponds to a transition/transversion (ts/tv) ratio of 1.5∶1. Interestingly, the observed ts/tv ratio is similar to the ratio obtained using RAD-seq method and the number of A/G substitutions almost equaled the number of C/T substitutions in the transitions class. 1,695 (96.6%) out of 1,753 loci were found suitable for genotyping in 52 multiplex assays (Table 1). 58 contigs containing SNPs were discarded as they failed to meet the specifications for assay design for genotyping.

A comparison of 454 and RAD-seq (Illumina) contigs revealed that two loci were common to both data sets (using mapping criteria of 95% identity and at least a stretch of 20 bp perfectly matching). These loci were not included in the assay design.

Out of the 1,144,367 reads obtained from individual assemblies for elephant A and B of *E. m. borneensis*, we identified 18,195 contigs (1.58% of all reads) that contained SSRs or microsatellites, 8,432 contigs obtained from elephant sample A, and 9,763 contigs from elephant sample B. 27,226 microsatellites (with ≥3 repeats) were identified in the 18,195 contigs, of which 18,188, 7,241, 1,471 and 326 contained mono-, di-, tri-, and tetra-nucleotide repeats, respectively (Table S2). The di- and tri-nucleotide microsatellite repeat classes were more abundant than the tetra-nucleotides. By increasing the number of repeats in our search from three to six, we observed a decrease in all repeat classes of microsatellites. For instance, the number of mono-nucleotide microsatellites decreased 2.6 fold, di-nucleotides 50 fold, tri-nucleotides 81 fold, and tetra-nucleotides 23 fold. The contigs containing classified microsatellites have been submitted to GenBank under accession numbers JX941469-JX941511. An estimation of the number of contigs containing suitable flanking sites for PCR amplification (for ≥3 repeats), gave a total of 1,706 loci for di-, tri-, and tetra-nucleotides. These contigs containing PAL represented 18.8% (1,706 out of 9,038 including only di-, tri-, and tetra-nucleotides) of the total number of classified microsatellites.

### Validation of Markers (SNPs and SSRs) Obtained from RAD-seq and 454 Shotgun Sequencing

The seven Bornean elephant samples (same samples that were used in the discovery panel of RAD-seq) were genotyped targeting 161 putative SNPs (124 from RAD-seq and 37 from 454) using five multiplex assays with 3–5 replicates per individual and generating 3,903 genotypes (obtained out of 4,340). 54% of calls in Sequenom were recorded as “conservative”, 15% “moderate”, 9% “aggressive”, 18% low probability (insufficient quality to make a call), 3% with no alleles and 1% were user calls (calls assigned by us). In addition, we assigned a ‘no-call’ to the genotype calls at 7 different loci in sample number 7 due to bad spectrum (non-predictable variation in peak height). 99% of the genotyped data successfully achieved a consensus. Seven genotypes were non-reproducible as they consistently failed to amplify and did not yield a consensus. Positive PCRs (number of PCRs giving correct or consensus genotype) were reliably obtained for all seven elephant samples with PCR success rate ranging from 83 to 94% (average across each sample) and 86–95% (average across each plex). The possible errors detected and resolved by repeated genotyping were mainly caused by: (1) allelic dropout leading to detection of false homozygotes in six elephant samples with highest allelic dropout rate observed in sample 1 (27%), (2) false alleles were detected in only one elephant (sample 3) and were in low proportion (0.16%). Table S3 provide details of observed genotyping errors for each multiplex assay (plex 1 to plex 5). Note that plex 1 to plex 4 (124 loci) represent assays developed for SNPs identified using RAD-sequencing and plex 5 (37 loci) represent SNPs identified through 454 shotgun sequencing. SNP genotyping error rates were compared to available data sets from another study in which SNPs were genotyped on human tissue samples using the same platform. The estimated genotyping error rate (false alleles) were found to be 0.11%–0.20% and all SNPs had allelic dropout rates of <0.5% [48]. Allelic dropout and false allele rates were found similar to those observed in our study. We detected 33 SNPs (20%) for which all Bornean elephants were heterozygotes, and were interpreted as the result of two co-amplifying monomorphic loci. When these 33 loci were evaluated in another Asian elephant subspecies (*E.m. indicus, n* = 3), 15 (45%) of these loci were also found as fixed heterozygotes. Of the remaining 18 loci, 11 loci were found as homozygote for one of the two alleles and were recorded with high confidence (a conservative call). For the remaining 7 loci, only one of the alleles (homozygote) was recorded with high probability. Further investigation on a large sample size is required to confirm the status of these duplicated loci in Asian elephants. Also, a blast search of these 33 contigs against *L. africana* genomic data produced significant hits for all except one. Twenty one loci (63%) aligned to two different positions in the same or different scaffolds of *L. africana* genome. Eleven loci (33%) showed unique alignment. It has been recognized that the BLAST algorithm can be rather inefficient in identifying homologous sequences when short contigs are involved, i.e. those originating from the Illumina platform [47]. For our population genetics analyses, these 33 loci were therefore split into 33 pairs of loci thus making a total of 194 loci. In total, out of these 194 loci, only 33 loci (17%) were found polymorphic in Bornean elephants (Table 1). Elephant sample 5 was the most successfully genotyped (100%) across all loci. Across all samples, average heterozygosity was estimated to be *H*_o_ = 0.057 respectively. Elephant sample 7 displayed observed heterozygosity (*H*_o_) higher than the other individuals (*H*_o_ = 0.10) (Table S3).

Out of 55 potential microsatellites, 20 primer pairs did not amplify on seven elephant samples and 10 produced unclear patterns in the electropherograms and hence discarded. Of the remaining 25 microsatellites (15 tetra-, 4 di-, 6 tri-microsatellites) producing scorable peaks, all were found to be monomorphic across the seven Bornean elephant samples tested.

As mentioned above, the *de novo* SNPs and microsatellites developed using both methods (RAD-seq and 454 sequencing) were also successfully genotyped on *E.m. indicus* samples (Table S3). Out of 194 loci, 12% (24) were found polymorphic in these three samples (Table 1). Out of the 25 microsatellite loci tested, six (2 di-, 4 tetra-nucleotides) displayed polymorphism, ranging from 2 to 4 alleles per locus.

### Characterization of Contigs from 454 and RAD-seq

All 454-contigs (100%) containing SNPs (*n* = 37) showed a significant blast match and were successfully mapped to different *L. africana* genomic positions (alignment positions found in 33 different scaffolds). In an effort to identify or predict the possible functions of the SNPs (only for the contigs with the best alignment) the analysis revealed that only 8% (3 out of 37) of the 454-contigs could be associated with one or more functions. The function was putatively associated to a SNP if the region surrounding the SNP, when blasted against *L. africana*, overlaps an *L. africana* gene, in which case a gene's GO (Gene Ontology) term (from Ensembl annotation) was associated to the SNP. If the contig is nearby a gene, but it does not overlap that SNP, no GO term was associated. That is why we have so few SNPs ‘annotated’. 24 unique GO terms (for *e.g*., protein binding, DNA repair, calcium ion transport etc.) were represented in these 454-contigs (Table S4). In the case of the RAD-seq contigs, 94.7% (491 out of 518 loci) produced a successful Blast hit against *L. africana* genome (alignment positions corresponding to 57 different scaffolds). For the remaining contigs, no significant matches were obtained using Blastn. 23% of SNP containing contigs (113 out of 491) could be associated with one or more GO terms (Table S5) and 35 unique GO terms were obtained (for *e.g*., sensory perception of smell, zinc ion binding etc.).

## Discussion

We are currently witnessing a revolution in the field of population and conservation genetics and molecular ecology due to the easy access to high throughput sequencing platforms, which have hastened the development of molecular markers [49]. Here, we used two different high throughput sequencing platforms to identify and test new genomic markers in the Bornean elephant, a non-model species. As noted in the introduction, the RAD-seq method has been successfully used to map genes in many studies in model species, such as *Drosophila* [50], *Neurospora* [51], barley [52] and *Caenorhabitis elegans* [53]. RAD-seq also proved useful in studies (including a few non-model species) aiming at investigating genome organization and population level analyses by developing high quality genomic markers, for species such as egg-plant [47], globe artichoke [54] and threespine stickleback [38]. This has also led to its successful and strong representation in many fish and crop plants [40]. However, the number of studies using RAD-seq is still limited and has only been applied to less than ten non-model taxa (see Table S6). Our study is thus one of the first of its kind where we explored RAD-seq to identify *de novo* markers in a non-model, highly endangered species with no prior genomic information and very low genetic diversity [42]. The number of SNPs (∼14,000) identified was quite high in a genetically depleted species which is on the same order as the numbers identified in other species for which samples were more readily available. For instance, RAD-seq resulted in identification of more than 45,000 SNPs in threespine stickleback [37], [38], ∼10,000 SNPs in egg-plant [47] and ∼34,000 in the globe artichoke [54]. Note that these numbers should not be taken at face value due to differences in the use of source of DNA, and sample size in the discovery panel. We believe that in our study a greater number of polymorphisms could have been further identified in the individuals used, as we found substantial variation in Illumina reads from the individual sample libraries. Other reasons were the use of related individuals in the discovery panel. Based on the keepers information two to three individuals probably belonged to the same social group. We observed low genetic diversity (average *H*_o_ = 0.057 across all samples) in Bornean elephants and 17% of the loci were polymorphic. Our results are in agreement with the previous study (and only available genetic data on this species) of Fernando *et al*., [42] who also reported low diversity (average *H*_e_ = 0.041 using five microsatellites). But further genotyping and investigation of these loci on a large number of elephants from different populations is required.

In theory, the number of RAD-tag fragments can be roughly predicted *in silico* assuming equal and random frequency of cut sites; but in practice, some restriction enzymes depart considerably from this expectation [55]. For instance, in this study we observed fewer RAD-tags (∼23,532–37,362) than predicted *in silico* (47,689) on the basis of the genome size of *L. africana* and its GC content. This discrepancy observed was most likely due to the quality of the samples, and the genomic DNA concentration.

Using the 454 sequencing system, we also identified a reasonably large number of SNPs (6,683) despite using low sequence coverage and only two individuals. Again, a significant number of polymorphisms in our samples may have been overlooked because of the stringent methodology used to declare SNPs. The advantage of the stringent filtering of the reads is that it provided a higher quality data set. Reads used for contigs assembly shared high sequence identity (around 97% mapped accurately) with the *L. africana* genome.

Previous attempts to identify SNPs in other non-model species using high throughput sequencing technology have been of two kinds: (1) full genome sequencing using very high level of coverage [56] which is still impractical for many non-model species, and (2) low coverage sequencing on several (>6) individuals coupled with the approach of generating a RRL library (reduced representation of the genome) [9]. The second approach remains popular and efficient as thousands of high quality SNPs were discovered even in the absence of an available genome sequence. For instance, using an RRL approach ∼ 150,000 SNPs were identified in mallard duck [36] and 20,000 SNPs in the great tit [9]. But RRL is limited to a few non-model species due to difficulty in obtaining samples for endangered species. But, the crucial point that must be stressed is that 454 sequencing scheme used here allowed us to identify SNPs with only 2 individuals from a species with low genetic diversity. This is very good news for conservation biologist working on species for which high-quality samples (blood, tissue) are difficult to obtain.

One of the major challenges as identified in our data was that RAD-seq yielded a high number of candidate SNPs with high coverage but a large proportion (around 75%) of the identified SNPs loci were found inappropriate for SNP assay design. This was due to their proximity to the ends of the large contigs and/or rarely due to the presence of other SNPs around them. Thus, in the absence of a reference genome the number of SNPs with sufficient flanking sequence for designing a genotyping assay remains limited [57]. On the other hand, the SNPs obtained from a 454 run were less numerous (6,683 SNPs in 1,753 contigs), located on longer contigs, and a large proportion, (96%) of them resulted in assay conversion rate. This also leads to the question of how comparable are our SNP validation rate with other studies. This is not easy to find as most of the studies report SNP detection in next-generation sequencing data from non-model species with limited, if any, experimental validation of discovered polymorphisms [28]. In this study, we gathered all of the available information on the discovery of markers and their validation data to make a comparison across different non-model species from a literature survey (Table S6). Note that our list is not meant to be exhaustive. As shown in this table, the conversion rate of validation assays was found to be variable among various non-model species. Much higher SNP validation rates (using Illumina Golden Gate) were observed in some species, such as ∼80% of SNPs were validated in different populations of teleost [8], 88.5% in common turkey [35], 94.7% in mallard duck [36] and 84% in the great tit [9]. Our high validation rate of ∼ 83–94% (for accurately genotyped SNPs identified using both shotgun, 454 and RAD-seq Illumina platform) is similar to the studies mentioned above and is also similar to those observed in other non-model species (particularly in fish and crop plants), where a RAD-seq was applied using same depth of coverage. For instance, successful SNP validation rate of 89.3% (using Illumina Golden Gate assay) in egg-plant [47] and 100% (using Sanger sequencing) in threespine stickleback [41] were reported (Table S6). Since different genotyping platforms were used in these studies for SNP validation, the results are once again not entirely comparable.

While our primary goal was the identification of SNP markers, we also identified SSR motifs using both approaches. However, there were important differences in the results. A much high number of microsatellites (7,241 di-, 1471 tri-, and 326 tetra-nucleotides) were identified using 454 sequencing with ∼18% of these loci which had primer design sites that could potentially be used to amplify and score microsatellite alleles based on length variation (repeat number). In the case of RAD-seq, 29% of loci (24 di-, and no tri-,-and tetra-nucleotides were identified) were found suitable for primer design. This indicates that the major drawback with high throughput sequencing approaches is the current technical limitation introduced by sequence read lengths [58], [59], [60]. Gardener *et al*., [61] isolated microsatellites using 454 sequencing from 27 vertebrate species and observed that the most abundant SSR motif type was di-nucleotide followed by tetra-, and tri-nucleotide repeat classes. Indeed, we also identified more di-nucleotides from 454 sequencing but the ratios of repeat classes (between mono-, and di-nucleotides) were very different (∼2.5 in 454 vs. ∼35 in RAD-seq). While microsatellites are normally expected to be more polymorphic than SNPs, this was not the case here as all newly found microsatellites were monomorphic. This may be due to a bias in the general approach since microsatellite polymorphism is correlated positively to repeat length and only short DNA stretches were identified. But we found that five of them were polymorphic in other Asian elephant sub-species (*E. m*. *indicus*) even though we only had 3 individuals. This again confirms that Bornean elephants have extremely low genetic diversity in comparison to other Asian elephant subspecies. Since these data on Bornean elephant were collected, the high throughput sequencing technologies have evolved significantly, most importantly with the improvement in Illumina read lengths (now up to 150 bases with the GAIIx) and accommodate paired-end sequencing from both ends of ∼200–600 bp fragments [32]. Similarly, 454 platforms have progressed significantly and will continue to improve, with a single GS FLX+ run already capable of generating 700 Mb of sequence data with an average read length of 700 bp [62].

Another potential issue related with the high throughput sequencing (also reported in other species) is the assembly of paralogous regions [8], [63], [64]. As observed in our validation assay 20% of the loci were labelled as potentially duplicated due to fixed heterozygosity in data sets from both RAD-seq and 454. Our ability to distinguish between paralogs and regions exhibiting similarity due to gene duplications and conversions remains limited. This also means that there may be additional markers which we identified as single locus but may in fact correspond to paralog regions. This could have happened if they were not fixed for different alleles as this would make their identification more difficult. Validation for all markers is therefore important to ensure that the identified SNPs are real single locus markers that can be used for genotyping. This issue could be overcome and minimized by combining paired-end sequencing with high coverage but again taking care of all these issues will also increase the cost of study [41].

In conclusion, our study (results from both sequencing methodologies) suggests that despite substantial differences in sample size, sequence throughput, read length, and average sequencing depth, similar results were obtained after the validation in terms of SNPs genotyping success rate. Our ability to identify thousands of genomic markers with relative ease will also make it possible to estimate many important parameters, related to conservation, such as inbreeding coefficients or relatedness. This will clearly allow conservation biologists to enter the new genomic era of non-model species.

## Methods

### Sampling and DNA Extraction

#### Ethics statement

Samples were collected by the veterinarians at Lok Kawi Wildlife Park and Oregon zoo during the routine health checks. Ethics approval was not required or sought for this research, as the sample were not collected specifically for this study. All samples from Sabah, Malaysia were exported under CITES (Convention on International Trade in Endangered Species of Wild Fauna and Flora) permit obtained from Sabah Wildlife Department.

### RAD Paired-end Sequencing Using Illumina Platform

Eight fresh whole blood samples (hereafter sample 1 to 8) were collected directly from Bornean elephants. Seven samples (sample 1 to 7) were collected from the Lok Kawi Wildlife Park, Sabah, Malaysia in January 2010 and one additional Bornean elephant sample (sample 8) was obtained from the Oregon zoo, Portland in the United States. Genomic DNA was extracted using DNeasy Blood & Tissue Kit (QAIGEN). 10–12 µg of gDNA was isolated from each sample at a concentration of 20 ng/ul that was quantified using Nanodrop, ND-1000 spectrophotometer. High quality genomic DNA is the most crucial aspect for the success of high throughput sequencing methods. Therefore, several rounds of DNA extraction were performed and pooled for the downstream applications.

### Whole Genome Shotgun Sequencing Using 454

Two Bornean elephant whole blood samples (hereafter sample A and B) were collected along the Kinabatangan River in Sabah, Malaysia in March 2009 (these two individuals were wild and different from those used in the RAD-seq method). DNA was prepared as mentioned above. The shotgun sequencing was performed at the Microarray and Next Gen Sequencing Facility at the Center for Applied Genetics and Technology, University of Connecticut, USA. Sample A and B were run separately in a 2-region PTP plate.

### RAD Paired-end Library Construction and Sequencing

The RAD library was constructed at Floragenex Inc. (Eugene OR, USA), according to the protocol described by Baird *et* *al.,* [37]. Genomic DNA (0.1–1 µg from each individual) was digested with the 5-methylcytosine sensitive restriction endonuclease, *Eag*I and processed into short DNA fragments or ‘tags’. The enzyme *Eag*I (recognition sequence, C*GGCCG; 6 bp) was selected for RAD profiling of the 8 elephant sample lines. DNA was digested for 60 min at 37°C in a 50 µL reaction containing 20 U of *Eag*I (New England Biolabs, Beverly MA, USA). The reactions were stopped by holding the temperature at 65°C for 20 min. The P1 adapter (a modified Illumina adapter, see [37]) was ligated to the products of the restriction reaction, and the “barcoding” of the various samples was achieved with a set of index nucleotides in the P1 adapter sequence. A 2.5 µL aliquot of 100 nM P1 adapter was added to each sample, along with 1 µL 10 mM ATP (Promega), 1 µL 10 × NEB Buffer 4.1 µL (equivalent to 1,000 U) T4 DNA ligase (Enzymatics, Inc) and 5 µL water, and the reaction was incubated at room temperature for 20 min, followed by heat-inactivation (20 min at 65°C). Thereafter, the reactions were pooled and the products randomly sheared to a mean size of 500 bp using a Bioruptor (Diagenode). The material was electrophoresed through a 1.5% agarose gel, and the DNA in the range 300–800 bp isolated using a MinElute Gel Extraction Kit (Qiagen). The DNA ends were treated with end blunting enzymes (Enzymatics, Inc) to remove overhangs, and the samples purified by passing through a MinElute column (Qiagen). 3′-adenine overhangs were then added by the addition of 15 U Klenow exo- (Enzymatics), followed by an incubation at 37°C for 10 min. Following re-purification, 1 µL 10 µM P2 adapter (a modified Illumina adapter, see [37]) was ligated, as described above for P1. The samples were then purified as above, and eluted in a volume of 50 µL. Following quantification (Qubit fluorimeter), 20 ng were taken as the template for a 100 µL PCR containing 20 µL Phusion Master Mix (NEB), 5 µL 10 µM P1 adapter primer (Illumina), 5 µL 10 µM P2 adapter primer (Illumina) and water. The PCR settings followed product guidelines (NEB) over 18 cycles. The amplicons were gel purified, the size range 300–700 bp was excised from the gel and its DNA content adjusted to 3 ng/µL.

A RAD library containing each sample was sequenced on a Genome Analyzer (GAIIx, Illumina, San Diego, CA) in two lanes using an asymmetric sequencing strategy: paired end 40 × 80 bp sequences were obtained for all 8 samples.

### RAD Sequence Analysis, Contig Assembly and SNPs Calling

The paired-end sequence reads from each sample were manually collected and segregated by barcode. Once samples were demultiplexed the quality and quantity of sequence obtain from each sample was assessed. We identified sample number ‘4’ as having the highest quality and quantity of sequence and therefore began our analysis with this sample. First, a set of unitag, or unique RAD single end sequences were identified with coverage ranging from 5x–500x. Next, all paired-end reads that were associated with each single-end unitag sequence obtained above were collected and, the program velvet [65] was used to assemble consensus contigs (∼150–500 bp) from the paired-end data. Paired-end sequencing enables sequencing of the two ends of the RAD-tags to be used for local *de novo* assembly [41]. The SNPs were called using a short read alignment algorithm which aligned paired-end non-assembled 80 bp Illumina reads from all elephant samples against the “elephant sample 4” assembly. In this manner genotypes were called using SAMtools [66] for each sample. Heterozygous SNPs were called in two different ways: (1) SAMtools uses a Bayesian caller to identify SNPs, and (2) Floragenex internal scripts use a threshold model to identify SNPs at a default rate of 7.5% alternate allele frequencies. Each SNP was assigned a designability score via a dedicated “assay design tool” defined for MassArray/TaqMan platform (http://www.illumina.com), which identified SNP loci free of other polymorphisms 60 bp either upstream or downstream. A quality score, based on the probability of good performance using the Illumina Golden Gate assay, was assigned to each SNP, where a score >0.6 indicated a high probability of success.

### Validation of SNPs

The identified contigs were used to design a group of multiplexed genotyping assays using the Sequenom MassArray Assay Design software (Sequenom, San Diego, CA, USA). To facilitate assay design, we screened the complete SNP panel to identify those alleles free of flanking polymorphisms (60 bp flanking SNPs in 5′ and 3′ direction, total window of 120 bp). Up to 40 different SNPs can be multiplexed in one assay, if primers are designed by the custom software to give unique mass range for each SNP. For contigs containing more than one SNP, only one SNP was selected per contig for validation. In addition, SNPs were discarded if the contig did not have at least 30 bp on either side of the SNP to allow for amplification. Multiplexes and primer sets are available from the first author upon request. Once the assays were identified, large-scale surveys of their frequencies were performed using the Sequenom iPLEX genotyping platform [67]. In this method, a short section of DNA containing a SNP is amplified from an individual sample by PCR. This is followed by a high-fidelity single-base primer extension reaction over the SNP being assayed, using nucleotides of modified mass. The different alleles therefore produce oligonucleotides with mass differences that can be detected using highly accurate Matrix-Assisted Laser Desorption/Ionization Time-of-Flight (MALDI-TOF) mass spectrometry. The software (SpectroTYPER) from Sequenom automatically translates the mass of the observed primers into a genotype for each reaction. The assay design was used for genotyping in a 384-well plate that included diluted *E.m. borneensis* genomic DNA samples (∼20 ng DNA /in 30 µl of water). For allele separation, the Sequenom MassARRAY™ Analyzer (Autoflex mass spectrometer) was used. Genotypes were assigned by the MassARRAY SpectroTYPER RT v3.4 software (Sequenom) based on the mass peaks present. The resulting data were manually inspected using SEQUENOM System Typer 4.0. The software uses a three parameter model to calculate significance of each putative genotype. A final genotype is called and assigned as ‘conservative’, ‘moderate’, ‘aggressive’, ‘low probability’, and ‘user call (manual calls)’ based on degree of confidence. SNPs were classified as “failed assays” when the majority of genotypes could not be scored due to low probability or when the samples did not cluster well according to genotype. SNPs that were out of Hardy-Weinberg Equilibrium (HWE) in one or more samples were cross-checked. Multiplex assays (plex 1 to plex 4) targeting 124 unique SNPs randomly selected from the set of identified SNPs were genotyped across a panel of seven elephants which are also the individuals used to discover the markers. Each elephant blood sample was amplified in 3–5 different replicates (average 4 replicates for each plex). After repeated genotyping, it is necessary to construct a consensus multilocus genotype (the most likely genotype based on all polymerase chain reaction (PCR) amplifications of a sample) and to estimate the genotyping error rates [68]. We used rigid criteria in scoring and accepting consensus genotypes in order to minimize potential genotyping errors [69], including the scoring of alleles and the validation by two persons independently. Therefore, we counted the number of positive PCRs (estimated error rates for a set of genotypes from repeated PCR) giving correct or consensus genotype. The proportion of genotyping errors, such as allelic dropout (when a heterozygote individual is genotyped as homozygote) and false alleles (when a homozygote individual is genotyped as heterozygote) was assessed using GIMLET v. 1.3.2 [70]. Analysis to estimate genetic diversity was performed using PEAS v1.0 (a Package for Elementary Analysis of SNP data) [71].

### 454 Library Preparation and Sequencing

500 ng of starting DNA was used in the 454 FLX shotgun library preparation, following the manufacturer’s protocol and quality control steps.

### 454 Sequence Assembly, Mapping and Marker Detection

Reads generated from whole genome shotgun sequencing of the two elephants (sample A and B) were aligned to generate a combined *de novo* assembly using the program GS Assembler v2.5.3 (454 Life Sciences). To explore data quality and relevance, we used draft genome sequence of African savanna elephant (*Loxodonta africana*) (loxAfr3; http://www.broadinstitute.org/ftp/pub/assemblies/mammals/elephant/, has been sequenced to 7x coverage, July 2009) to map the reads. A recent study by Rohland *et al*., [72] (using coalescent estimates) determined the range for the split between *Loxodonta* and Eurasian elephantids as 4.2–9 Ma (million years). For SNP prediction, reads from the two elephant samples were then mapped to the combined assembly contigs using the Burrows-Wheeler Aligner (BWA) and the SW algorithm longer reads [73]. Data was processed to identify the SNPs between the reference and reads from the two Bornean elephant samples. Mapping results were then processed in SAMtools to build and filter ‘pileup’ files. Alignment of the short reads and consensus base calls were made using the MAQ software [66] and results were filtered based on SNP quality scores where only variable sites were reported. SNPs were positively identified, if SNP quality scores was greater than or equal to 20 or, if identify as an indel, greater than or equal to 50. SNP genotyping was performed only for a single set of identified multiplex assay (plex 5) consists of 37 independent SNPs.

### Microsatellite Detection and Primer Designing

8,432 contigs and 9,763 contigs from assemblies for elephant samples A and B, respectively, were converted into single FASTA format files. To develop a set of about 20–30 microsatellite markers, we conducted several screenings with different search criteria, i.e. using different minimum repeat lengths (between 3 and 6 for di-, tri-, and tetra-nucleotides) in order to extract a total of 50–100 sequences with microsatellite repeats using MSATCOMMANDER version 0.8.1 [74]. This software has an inbuilt workflow that enables the simultaneous detection of repeat motifs. We were interested in targeting small DNA product size (100–200 bp) to apply these markers on non-invasive elephant samples. The primers were designed manually using the software OLIGO version 3.4 [75]. The contigs chosen were the ones with the longest reads to get the best primer options. If the microsatellite repeat is detected too close to the extremity of the contig, the locus is discarded. We screened contigs with microsatellite loci for flanking regions with high quality PCR priming sites; we refer to such loci as ‘potentially amplifiable loci’ or PAL. We designed primers for only a subset of PAL (with ≥4 repeats for di-, tri-, and tetra-nucleotide classes). 55 primers were designed (12 di-, 10 tri-, and 33 tetra-nucleotides) and were tested in the lab.

### Validation of Microsatellites

The PCR amplified products were sequenced to confirm the microsatellite repeat before fluorescent tagging. Variability of microsatellite loci was analyzed by PCR using a 5′-fluorescence-labelled forward primer by using 20–30 ng of genomic DNA as template. PCR was performed in a total volume of 10 µL, containing 10 mM Tris-HCl, pH 9.0, 50 mM KCl, 2 mM MgCl_2_, 0.2 mM of each dNTP, 0.2 mM of both forward and reverse primer, and 0.75 U *Taq* polymerase (QBIOgene). Amplifications were performed using touchdown PCR in a BioRad (My Cycler) thermocycler with the following reaction profile: initial denaturation at 95°C for 3 min, 15 cycles: 95°C for 30 s, an elevated locus-specific annealing temperature *T*a +7.5°C for 45 s, 72°C for 1 min; 20 cycles: 95°C for 30 s, *T*a°C for 45 s, 72°C for 1 min; and a final extension at 72°C for 5 min. Fragment size was determined on a 3130 Genetic Analyzer (Applied Biosystems) multicapillary automatic sequencer using the GENEMAPPER version 3.7 and an internal size standard (ROX 500, Applied Biosystems). The newly designed microsatellite primers for di-, tri-, and tetra-nucleotide loci were screened on seven Bornean and three other Asian elephant samples (*E. m. indicus*) and genotypes were recorded.

### Characterization of the Contigs from 454 and RAD-seq (Illumina)

Contigs containing potential SNPs including those validated by genotyping (*i.e*., 37 from the 454 run and 518 from RAD-seq) were used for Blastn searches and gene ontology (GO) annotation. Also, a precise blast was performed by using FASTA files containing just short sequences of 120 bp SNP flanking sequences to determine if the putative SNPs were associated with genes, not to provide a complete annotation of these sequences. All contigs were queried against the sequence database of *L. africana* genome (Ensembl genome assembly for *L*. *africana*) using NCBI Basic Local Alignment Search Tool (BLAST+, version 2.2.26+). The gene ontology annotation files linking the gene ontology terms to the Ensembl *L. africana* gene identifiers were obtained from biomart (http://www.biomart.org). The contigs from Bornean elephant were annotated with the gene ontology terms associated with the orthologous *L. africana* gene. The Blastn searches were performed using e-value cut-off of 10^−5^ and the best alignment match and overlapping positions were noted.

## Supporting Information

Table S1 **List of RAD tags/loci with genotypes.** A complete list (vcf file) of all RAD tag/loci where genotypes were obtained for all individuals (100%) for 1,200 loci/RAD tags, and for 75% of the individuals (i.e. six or more) for 1,700 loci. For the remaining 400 loci, the SNPs were shared by less than six individuals. Eight *E. m. borneensis* samples were used in the discovery panel of RAD-sequencing using Illumina platform.
(XLSX)

Table S2 **Summary of SSRs (simple sequence repeats) identified in the Bornean elephant sequence dataset.** The number of SSRs or microsatellite loci identified in *E. m. borneensis* (*n* = 2) using the 454 shotgun sequencing approach, and the subset of these that are potentially amplifiable (containing suitable PCR priming sites) and validated. The di- and tri-nucleotide microsatellite repeat classes (≥3) were more abundant than the tetra-nucleotides.
(XLSX)

Table S3 **Estimation of genetic diversity and genotyping error rates.** Comparison of observed heterozygosity (*H*_o_), missing genotypes, allelic dropout, number of positive PCRs, false alleles, and mean number of allele (MNA) for the genotyped samples of *E. m. borneensis* (*n* = 7) and *E. m. indicus* (*n* = 3). Multiplex assays (five plexes) targeting 194 unique SNPs were validated across all elephant samples using Sequenom iPLEX platform. Note that plex1 to plex 4 (124 loci) represent assays developed for SNPs identified using RAD-sequencing and plex 5 (37 loci) represent SNPs identified through 454 shotgun sequencing (see Methods).
(DOC)

Table S4 **Gene Ontology (GO) term representation of 454-contigs.** The GO terms were obtained for 454 contigs containing SNPs using Ensembl gene annotation of *L. africana.* In these 454-contigs, 24 unique GO terms were represented.
(XLSX)

Table S5 **Gene Ontology (GO) term representation of RAD-seq contigs.** The GO terms were obtained for RAD-contigs containing SNPs using Ensembl gene annotation of *L. africana.* In these RAD-seq contigs, 35 unique GO terms were represented.
(XLSX)

Table S6 **Recent SNP discovery efforts in non-model species.** A literature review of recent SNP discovery efforts in non-model species using high throughput sequencing platforms.
(XLSX)
