# Correction: Two Different High Throughput Sequencing Approaches Identify Thousands of *De Novo* Genomic Markers for the Genetically Depleted Bornean Elephant

**DOI:** 10.1371/annotation/8aaa2a9c-9c15-4214-808d-db8b0bd8ab88

**Published:** 2013-07-09

**Authors:** Reeta Sharma, Benoit Goossens, Célia Kun-Rodrigues, Tatiana Teixeira, Nurzhafarina Othman, Jason Q. Boone, Nathaniel K. Jue, Craig Obergfell, Rachel J. O'Neill, Lounès Chikhi

The following statement is missing from the Funding Information:

NJ, CO, and RO were supported by the National Science Foundation and the Center for Applied Genetics and Technology at UCon.

