# Supplementary material for: Two Different High Throughput Sequencing Approaches Identify Thousands of De Novo Genomic Markers for the Genetically Depleted Bornean Elephant
Source: PLoS One. 2012 Nov 21;7(11):e49533. doi: 10.1371/journal.pone.0049533 (PMC3504023; doi:10.1371/journal.pone.0049533)
Supplement: Table S3 — Estimation of genetic diversity and genotyping error rates. Comparison of observed heterozygosity (H o), missing genotypes, allelic dropout, number of positive PCRs, false alleles, and mean number of allele (MNA) for the genotyped samples of E. m. borneensis (n = 7) and E. m. indicus (n = 3). Multiplex assays (five plexes) targeting 194 unique SNPs were validated across all elephant samples using Sequenom iPLEX platform. Note that plex1 to plex 4 (124 loci) represent assays developed for SNPs identified using RAD-sequencing and plex 5 (37 loci) represent SNPs identified through 454 shotgun sequencing (see Methods). (DOC) [file pone.0049533.s003.doc]

*No calls were assigned at 7 different loci in this sample due to bad spectrum (non-predictable variation in peak height)
